# MULTI-OMIC DISSECTION OF ALZHEIMER’S DISEASE: DISENTANGLING AGING TRAJECTORIES, NEURODEGENERATION AND PATHOPHYSIOLOGICAL CONFOUNDERS

**DOI:** 10.1093/geroni/igaf122.1247

**Published:** 2025-12-31

**Authors:** Agustin Ruiz Laza, Sudha Seshadri

**Affiliations:** Glenn Biggs Institute for Alzheimer's and Neurodegenerative Diseases, San Antonia, Texas, United States; Glenn Biggs Institute for Alzheimer's and Neurodegenerative Diseases, San Antonia, Texas, United States

## Abstract

Alzheimer’s disease (AD) is a heterogeneous, age-related neurodegenerative condition in which genetic risk, exposome, and disease biology interact in complex and non-linear ways. Multi-omic technologies now allow us to dissect this complexity at scale. We integrate genomics, CSF proteomics (SomaScan 7k), CSF lipidomics (LC–MS/MS), ancillary CSF measurements, and clinical phenotyping across deeply characterized individuals to identify latent molecular axes that shape CSF composition. Two orthogonal components — CSF turnover and blood–brain barrier integrity — explain ∼75% of CSF omic variance and drive strong artifactual inflation in biomarker signatures if not accounted for. Adjustment for these cryptic biological states improves classification performance, reveals more faithful disease-linked molecular signals, and allows a clearer separation of natural aging and disease trajectories. Our data show that multi-omic integration is not only descriptive — it is necessary to deconvolve the causal layers that structure AD phenotypes. This supports a precision therapeutics agenda in which molecular endophenotypes become the operative unit for disease modeling, biomarker development, and targeted intervention.

